# Comparison of CT referral justification using clinical decision support and large language models in a large European cohort

**DOI:** 10.1007/s00330-025-11608-y

**Published:** 2025-04-27

**Authors:** Mor Saban, Yaniv Alon, Osnat Luxenburg, Clara Singer, Monika Hierath, Alexandra Karoussou Schreiner, Boris Brkljačić, Jacob Sosna

**Affiliations:** 1 School of Health Sciences, Faculty of Medical and Health Sciences, Tel Aviv University, Tel Aviv, Israel; 2 The Gertner Institute for Epidemiology and Health Policy Research, Sheba Medical Center, Tel Hashomer, Ramat-Gan, Israel; 3 Medical Technology, Health Information and Research Directorate, Ministry of Health, Jerusalem, Israel; 4 European Society of Radiology, Vienna, Austria; 5 Radiation Protection Department, Health Directorate, Ministry of Health, Luxembourg City, Luxembourg; 6 Department of Radiology, University Hospital Dubrava, University of Zagreb School of Medicine, Zagreb, Croatia; 7 Department of Radiology, Hadassah Medical Center, Faculty of Medicine, Hebrew University of Jerusalem, Jerusalem, Israel

**Keywords:** Tomography (X-ray computed), Decision support systems (clinical), Artificial intelligence, Referral and consultation, Guideline adherence

## Abstract

**Background:**

Ensuring appropriate use of CT scans is critical for patient safety and resource optimization. Decision support tools and artificial intelligence (AI), such as large language models (LLMs), have the potential to improve CT referral justification, yet require rigorous evaluation against established standards and expert assessments.

**Aim:**

To evaluate the performance of LLMs (Generation Pre-trained Transformer 4 (GPT-4) and Claude-3 Haiku) and independent experts in justifying CT referrals compared to the ESR iGuide clinical decision support system as the reference standard.

**Methods:**

CT referral data from 6356 patients were retrospectively analyzed. Recommendations were generated by the ESR iGuide, LLMs, and independent experts, and evaluated for accuracy, precision, recall, F1 score, and Cohen’s kappa across medical test, organ, and contrast predictions. Statistical analysis included demographic stratification, confidence intervals, and *p*-values to ensure robust comparisons.

**Results:**

Independent experts achieved the highest accuracy (92.4%) for medical test justification, surpassing GPT-4 (88.8%) and Claude-3 Haiku (85.2%). For organ predictions, LLMs performed comparably to experts, achieving accuracies of 75.3–77.8% versus 82.6%. For contrast predictions, GPT-4 showed the highest accuracy (57.4%) among models, while Claude demonstrated poor agreement with guidelines (kappa = 0.006).

**Conclusion:**

Independent experts remain the most reliable, but LLMs show potential for optimization, particularly in organ prediction. A hybrid human-AI approach could enhance CT referral appropriateness and utilization. Further research should focus on improving LLM performance and exploring their integration into clinical workflows.

**Key Points:**

***Question*** *Can GPT-4 and Claude-3 Haiku justify CT referrals as accurately as independent experts, using the ESR iGuide as the gold standard?*

***Findings*** *Independent experts outperformed large language models in test justification. GPT-4 and Claude-3 showed comparable organ prediction but struggled with contrast selection, limiting full automation.*

***Clinical relevance*** *While independent experts remain most reliable, integrating AI with expert oversight may improve CT referral appropriateness, optimizing resource allocation and enhancing clinical decision-making.*

**Graphical Abstract:**

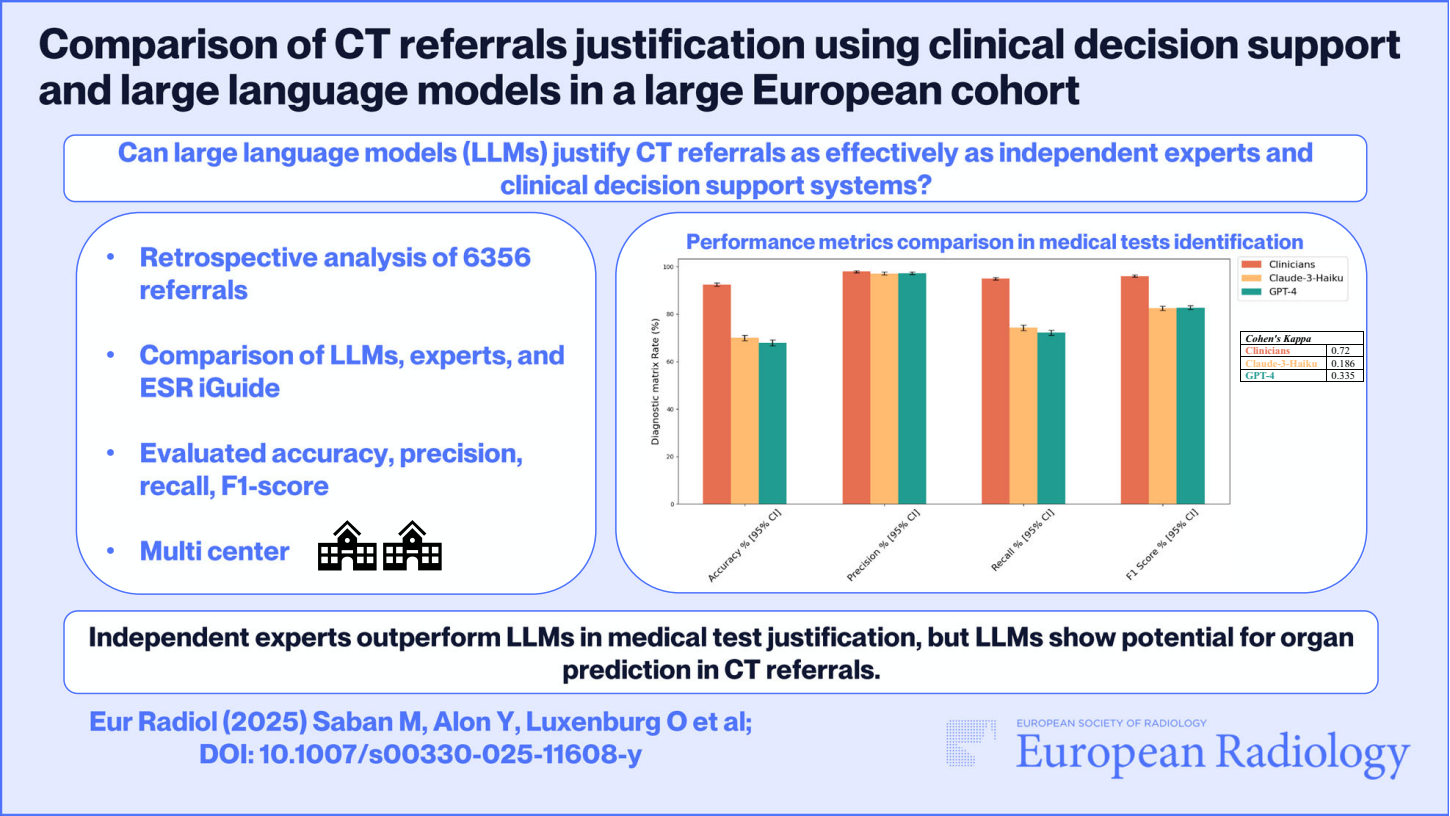

## Introduction

Ensuring the appropriate use of diagnostic imaging, particularly computed tomography (CT) scans, is a critical concern in modern healthcare. CT exams contribute the largest portion of radiation exposure from medical sources [1, 2]. Accordingly, there is growing recognition of the need to justify each exam based on clinical necessity [3–6]. Retrospective studies have found inappropriate CT use rates ranging from 10 to 39% when referrals are assessed against clinical guidelines [3, 7, 8]. As overall CT utilization continues to rise rapidly due to technological advances as well as expanding populations and indications, optimizing the appropriateness of each exam becomes increasingly vital. This is crucial for patient safety while delivering clinical benefits [4, 9–11].

In recent years, large language models (LLMs) have shown promise in assisting radiologists and clinicians with a variety of tasks [12–16]. These artificial intelligence (AI) powered systems can analyze a patient’s clinical history, symptoms, and imaging data to provide personalized recommendations on the most appropriate diagnostic tests and imaging modalities. By doing so, LLMs can enhance adherence to evidence-based guidelines, reduce inappropriate imaging utilization, and improve the overall efficiency of radiology workflows. Moreover, they can alleviate the growing workload faced by radiologists by automating repetitive tasks, allowing clinicians to focus on complex cases requiring human expertise. This optimizes resource utilization and supports better patient outcomes [17]. However, successful integration of AI into clinical workflows requires addressing challenges, such as ensuring interpretability, mitigating bias, and complying with regulations like the General Data Protection Regulation (GDPR) [17–20].

Decision support tools, such as the European Society of Radiology’s iGuide (ESR iGuide) clinical decision support system (CDSS), have been developed to facilitate clinical decision-making for CT scan referrals based on appropriateness criteria [21–23]. The ESR iGuide was developed in cooperation with the American College of Radiology (ACR) and represents Europeanized ACR Appropriateness Criteria, which are evidence-based guidelines developed through multiple expert consensus panels [24, 25]. However, research evaluating the clinical implementation and success of such decision support tools, as well as the real-world performance of the ESR iGuide in assisting diagnostic decision-making and optimizing medical resource utilization on national scales, has been limited to date [23, 26].

Advancing the use of decision support systems, including both clinical guidelines and emerging AI-based technologies like LLMs, is directly aligned with the goal of the 2021 EU-JUST-CT project to coordinate efforts improving CT justification in Europe [1, 27]. The aim of this study was accordingly to evaluate the performance of LLM-based recommendation systems, including Generative Pre-trained Transformer 4 (GPT-4) and Claude-3 Haiku, in assisting clinicians with CT referral justification, in comparison to independent experts’ assessments.

## Materials and methods

Study approval was obtained from the local research ethics committees or Institutional Review Boards (IRBs) at each participating imaging facility. At each site, the IRB either approved the study or waived the requirement for informed consent due to the retrospective nature and de-identification of the data.

### Data collection and recommendations

CT referral data was retrospectively extracted from the electronic medical records and imaging systems of 6356 patients treated in various medical centers [28]. Patient data was collected including age, gender, patient status, clinical background, clinical indications, and referral specialties.

Independent expert assessments were gathered focusing on consecutive CT examination referrals conducted over one to two workdays in 2022. Each referral was evaluated by board-certified radiologists at the participating centers, who regularly perform clinical assessments of CT referrals as part of their daily practice. These evaluations thus reflected real-world clinical decision-making across diverse healthcare settings in Europe. Recommendations were generated using three systems: (1) ESR iGuide assessments, conducted by two radiologists for each referral with arbitration to resolve disagreements, referred to as the reference standard for this study, (2) Open AI GPT-4, and (3) Anthropic’s Claude-3 Haiku.

### ESR iGuide

The ESR iGuide was utilized to evaluate the appropriateness of imaging test referrals. Two radiologists reviewed each referral and input the relevant patient data into the ESR iGuide portal, which provided recommendations on a scale from 1 (not recommended) to 9 (highly recommended). An arbitrator evaluated the appropriateness scores when the difference between the radiologists was more than 2. The output of this system was used as the true value, aka the reference standard.

### Large language models

We chose two state-of-the-art generative AI models to generate recommendations based on patient data, accessed via the models’ application programming interfaces (APIs). The first one, GPT-4, is described by OpenAI as a “large multimodal model capable of solving difficult problems with high accuracy due to its advanced reasoning capabilities and broader general knowledge.” GPT-4 has a context window of 128k tokens, a maximum output of 8192 tokens, and a knowledge cutoff date of September 2021 [29]. The second one, Claude-3-Haiku, is the fastest of the three versions of Anthropic Claude-3, with a context window of 200k tokens, a maximum output of 4096 tokens, and a knowledge cutoff date of August 2023 [30]. We used the same settings of 0.1 temperature and the same prompt set for both models to ensure consistency. Moreover, we consciously assessed the models’ foundational medical knowledge and decision-making abilities without offering external guidelines or contemporary medical protocols. This strategy enabled us to examine how effectively the models’ inherent knowledge corresponded with current clinical practices while acknowledging the specific cutoff dates of their training data reported by Anthropic [31] and OpenAI [32] by the date of the study. We aimed to gain insights into the models’ baseline performance in recommending imaging studies based exclusively on their pre-existing knowledge. All patient data underwent thorough preprocessing to guarantee complete anonymization per our ethical guidelines and GDPR requirements. For each clinical case, the input data included standardized fields comprising referring specialty, patient demographics (age and sex), patient status, clinical background, and the rationale for the examination or clinical indication, all integrated into the prompt.

Our prompt development followed a systematic approach to ensure a fair comparison with clinical practice, addressing some initial findings from our preliminary testing. We discovered that unconstrained prompts led LLMs to suggest MRI scans in 15–20% of cases, which posed a methodological challenge since our dataset exclusively contained CT referrals. This reflected real-world clinical decisions where CT was deemed the appropriate modality. To address this, we modified the prompt rationale, recognizing that our dataset specifically comprised cases where clinicians had determined CT as the relevant examination. Allowing MRI recommendations would have created an artificial comparison scenario and unfairly disadvantaged the LLMs’ performance metrics relative to clinical practice.

Consequently, we implemented a final prompt: “Based on the following patient details, provide a recommendation for an exam in the format, without explanation: ‘Exam, organ, with or without IV contrast.’ Note: MRI machines are not available for test recommendations.” This modification effectively aligned the LLMs’ decision space with the real-world constraints reflected in our dataset, reducing MRI recommendations to nearly 0% and enabling a more accurate comparison with clinical outcomes.

The average generation time per data row for each LLM was approximately 1.4 s for GPT-4 and about 1.2 s for Claude-3-Haiku.

### Recommendations processing and labeling

**Fig. 1:**
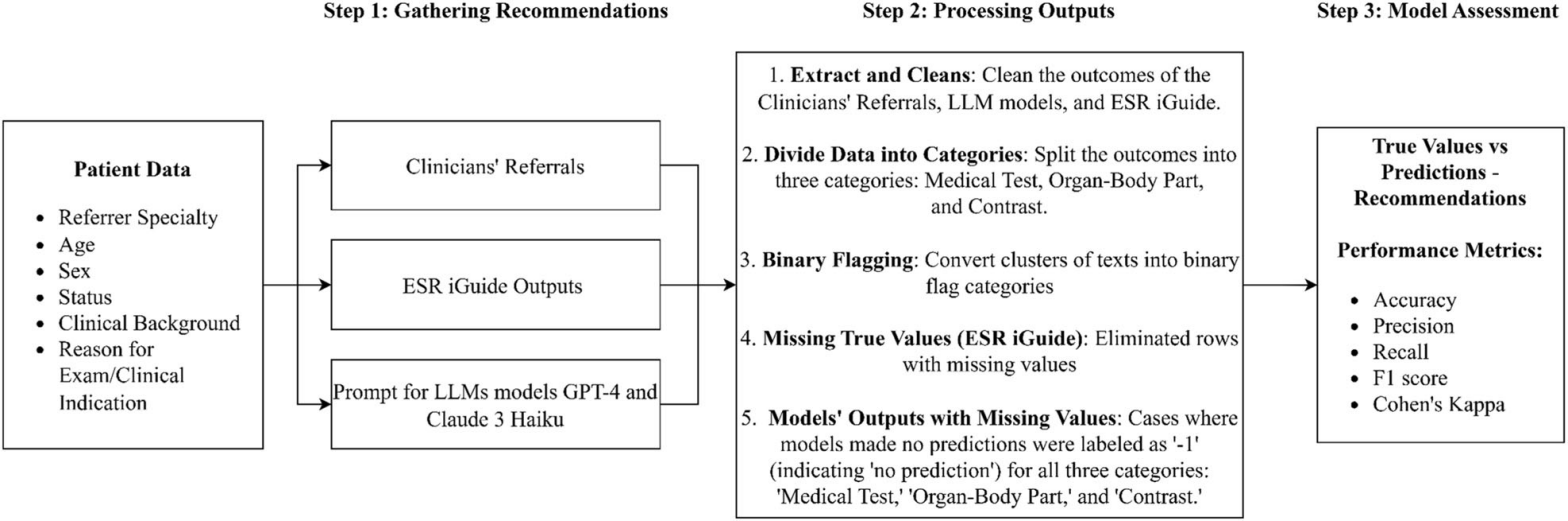
Overview of medical recommendation and assessment process

The independent experts’ assessments encountered several challenges due to missing or inconsistent data. These issues included referrals that lacked specified body parts, body parts without corresponding tests, typographical errors, and ambiguous notations. To systematically address these issues, we categorized the data into three major components: “Medical Test,” “Organ-Body Part,” and “Contrast.” Each component was processed independently to mitigate inconsistencies and ensure a more structured evaluation. This approach allowed for a targeted analysis of model performance across distinct elements of CT referrals (Fig. 1). The data preprocessing pipeline also incorporated robust cleaning and labeling protocols to minimize ambiguity and missing information. Ambiguous entries were flagged and either clarified through consensus among experts or excluded from analysis if no resolution was possible. This ensured the final dataset used for evaluation was as accurate and consistent as possible.

### Data cleaning and formatting

Quantifying the three major categories “Medical Test,” “Organ-Body Part,” and “Contrast” was challenging due to diverse entries and missing values ranging from 5 to 20%. Nevertheless, the reference standards were documented for nearly all entries (*n* = 5508, 86.65%), allowing us to evaluate the performance of independent experts’ assessment versus the LLM models using these features. The data cleaning and formatting were handled using a custom Python class. Key functionalities included the following:

#### Cleaning recommendations

The cleaning process for raw recommendations involved removing special characters and standardizing punctuation, normalizing text cases, standardizing medical abbreviations, eliminating extraneous spaces, and resolving ambiguities through expert consensus.

#### A labeling strategy

For each category, we removed rows with missing values as per the reference standard. This resulted in *n* = 5450 rows being eligible for evaluation in the Medical Tests category, *n* = 5448 in the Organs category, and *n* = 5340 in the Contrast category. Consequently, the data loss was approximately 1.05% for Medical Tests, 1.09% for Organs, and 3.05% for Contrast. In cases where the independent experts’ assessment or the outputs from the LLMs contained missing values, we designated these outputs with a label of ‘−1’ across all categories (Medical Test, Organ-Body Part, and Contrast). This labeling strategy allowed us to differentiate between situations where no recommendation was made and where specific recommendations were provided. This distinction was proven particularly crucial in the Contrast category, where independent expert assessments lacked contrast protocol prescriptions for 48.6% (*n* = 2595) of the data entries eligible for evaluation.

### Model performance

We evaluated the performance of the models separately for each category using the following metrics:

#### Accuracy

This measured the proportion of correct predictions among the total number of cases in comparison to the reference standard. Relying solely on accuracy can be misleading in scenarios with class imbalance, often seen in clinical datasets.

#### Precision

This was the ratio of true positive predictions to the total number of positive predictions (true positives and false positives). The precision metric thus assessed the models’ proficiency in minimizing false positives.

#### Recall

This was the ratio of true positive predictions to the total number of actual positives (true positives and false negatives). This metric thus evaluated the models’ ability to identify all relevant cases, which is crucial in clinical scenarios where missing a diagnosis could have serious consequences.

#### F1 score

This measured the harmonic mean of precision and recall, providing a balance between Precision and Recall. It is especially useful in cases where there is an uneven class distribution or when both false positives and false negatives are important to consider.

#### Cohen’s kappa

This metric assessed how well the models’ predictions aligned with the true values, considering the possibility of agreement occurring by chance. Cohen’s Kappa evaluates the likelihood of such a chance agreement, which is particularly important when comparing predictions to expert annotations. Furthermore, it provides a more conservative and reliable assessment of agreement than simple accuracy, especially in cases where some categories are more dominant. We calculated the metrics above by converting bitmasks to binary indicator arrays to handle multilabel data, performing bootstrapping to estimate confidence intervals, and applying weighted averaging.

We used binary encoding schemes to standardize three key aspects of imaging recommendations. A bitmask is a sequence of bits (0 s and 1 s) representing various states or categories, where each bit corresponds to a different category or class. We implemented three distinct bitmask systems: a 10-bit encoding for anatomical regions (head, neck, thorax, abdomen/pelvis, upper extremities, lower extremities, spine, skeletal system, lymphatic system, and body), a 3-state encoding for contrast usage (without IV contrast, with IV contrast, and with or without IV contrast), and an 8-bit encoding for medical test types (CT Scans, MRI, Ultrasound and Doppler Studies, Radiography and X-Ray, Nuclear Medicine, Invasive and Interventional Procedures, Cardiovascular Specific Exams, and Health Check-ups).

We determined the number of classes based on the maximum value in each category’s dataset to convert these bitmasks to binary indicator arrays. We converted each bitmask value to a binary string of the appropriate length. This binary string was then converted to an array of integers, where each element was either 0 or 1, indicating the absence or presence of a class. This method was particularly useful in our study as it allowed us to simultaneously capture multiple recommendations across anatomical regions, contrast requirements, and test types in a single case.

Bootstrapping is a statistical technique used to estimate the distribution of a metric by repeatedly sampling from the data with replacement. It was appropriate to apply here since it provided robust estimates of confidence intervals for metrics like accuracy, precision, recall, and F1 score. We then employed a weighted averaging approach for multilabel evaluation to balance the importance of individual class performance with the overall instance performance. Combining these methods ensured that the performance metrics accurately reflected the models’ capabilities and provided a reliable assessment of their performance in clinical decision-making contexts. We used chi-square tests to compare performance between systems (clinicians, Claude-3-Haiku, GPT-4) across all three evaluation tasks (Medical Tests, Organs, Contrast). Statistical significance was set at *p* < 0.05.

### Ethical considerations

In addition to the IRB approvals mentioned earlier, strict procedures were instituted to anonymize all patient data in accordance with the GDPR to meet data privacy standards. Moreover, the strictest standards were applied to ensure secure data collection, processing, and sharing that adhered to the GDPR principles of lawfulness, fairness, transparency, and purpose limitation.

We utilized Python 3.11 for all data analysis and processing tasks. This included employing various libraries such as Pandas for data manipulation, NumPy for numerical operations, and Scikit-learn for machine learning model implementation and evaluation.

## Results

### Patient sample

The study sample consisted of 6356 patients with a wide spectrum of medical conditions and clinical scenarios, reflecting the diverse patient population (Table 1).

**Table 1:** Descriptive statistics

Age mean ± Std	63.39 ± 16.9
Sex	
Male, *N* (%)	3164 (49.78)
Female, *N* (%)	3159 (49.7)
Patient status	
Emergency, *N* (%)	402 (6.72)
Inpatient, *N* (%)	1432 (23.95)
Outpatient, *N* (%)	3624 (60)
Unknown, *N* (%)	522 (8.73)

Descriptive statistics for patient characteristics among countries represented in the collected data, *n* = 6356

### Performance on medical tests

**Fig. 2:**
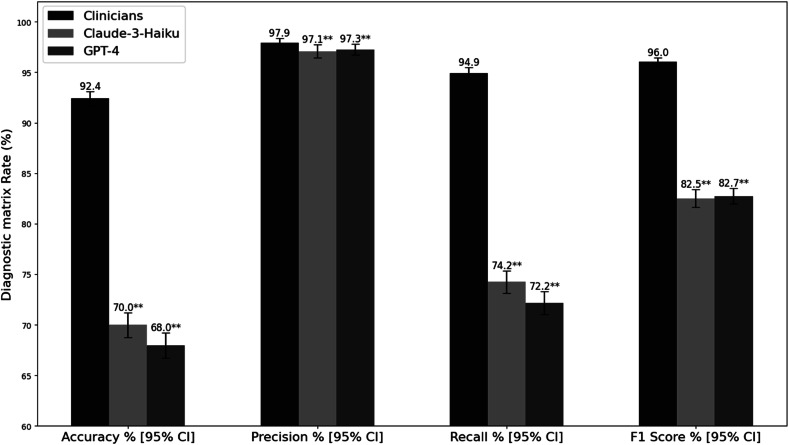
Performance metrics comparison in medical tests identification. Error bars represent 95% confidence intervals calculated using bootstrapping with 1000 resamples. Statistical significance was determined through comparison with clinicians’ evaluations: *p < 0.05, **p < 0.01, ***p < 0.001

There were challenges in cleaning and transforming data entries from independent experts’ assessments into a reliable source, and there was also a diversity of independent experts’ assessments from across Europe. Their medical assignment performance showed varying levels of superiority compared to Claude-3-Haiku and GPT-4 across different performance metrics for medical tests (*p* < 0.01), with Cohen’s Kappa values of 0.72 for clinicians, 0.186 for Claude-3-Haiku, and 0.335 for GPT-4 (see Fig. 2).

### Performance on organ-body part predictions

**Fig. 3:**
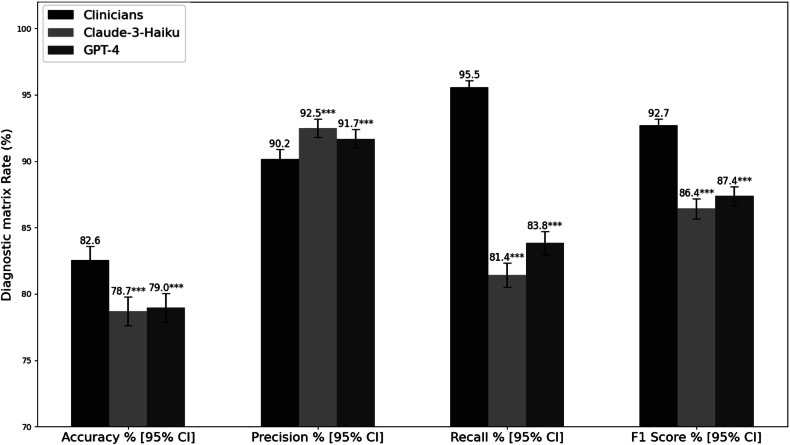
Performance metrics comparison in organ-body part identification. Error bars represent 95% confidence intervals calculated using bootstrapping with 1000 resamples. Statistical significance was determined through comparison with clinicians’ evaluations: *p < 0.05, **p < 0.01, ***p < 0.001

When evaluating performance concerning organs and body parts, the data suggested that while the independent experts’ assessment maintained a lead in overall performance (*p* < 0.001), the Claude-3-Haiku and GPT-4 models performed comparably well, especially in terms of accuracy and precision (Fig. 3). The level of agreement evident in the kappa values (0.786 for independent experts, 0.732 for GPT-4, and 0.725 for Claude-3-Haiku) indicated that both of the LLM models demonstrated reliable performance in identifying organ-body parts, although they still fell short of matching human expert levels.

### Performance on contrast predictions

**Fig. 4:**
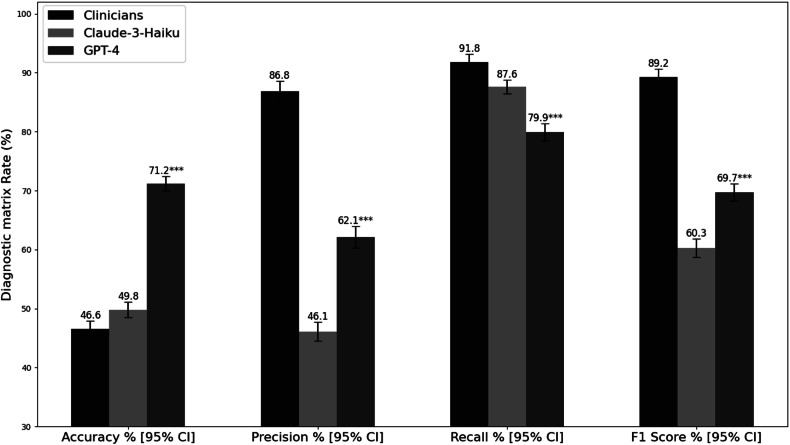
Performance metrics comparison in contrast protocol identification. Error bars represent 95% confidence intervals calculated using bootstrapping with 1000 resamples. Statistical significance was determined through comparison with clinicians’ evaluations: *p < 0.05, **p < 0.01, ***p < 0.001

In the contrast category (see Fig. 4), GPT-4 showed higher accuracy at 71.2% (95% CI: 69.97–72.4%), surpassing both independent experts’ assessments at 46.6% (95% CI: 45.26–47.93%) and Claude-3-Haiku at 49.81% (95% CI: 48.47–51.15%) (*p* < 0.001). This performance is reflected in GPT-4’s Cohen’s kappa of 0.457, indicating moderate agreement with the reference standard; substantially higher than the independent experts’ assessment of 0.308 and Claude-3-Haiku’s assessment of 0.041. The label distribution clearly illustrated the discrepancies between the reference standard labels and Claude-3-Haiku’s predictions. For the reference standard, the labels were as follows: ‘With IV contrast’—2370, ‘Without IV contrast’—2657, and ‘With or without IV contrast’—312. In contrast, Claude-3-Haiku predictions showed ‘With IV contrast’—4962, ‘Without IV contrast’—159, ‘With or without IV contrast’—53, and ‘No predictions’—165. This significant mismatch contributed to the overall disagreement between Claude-3-Haiku’s recommendations and the reference standard, as evidenced by the low Cohen’s kappa value. This contrasted with other categories where Claude-3-Haiku showed high precision and lower recall, resulting in better agreement with true values.

## Discussion

The results of this study provide valuable insights into how LLMs compare to independent experts’ assessments in CT referral justification. Independent experts consistently outperformed the LLMs across all categories and metrics, particularly in medical test justification. This underscores the continued importance of clinical judgment and specialized medical knowledge in navigating the complexities of real-world referral data, which often contains inconsistencies or incomplete information [20, 33]. The robust performance of independent experts, despite inconsistencies in referral data, highlights their ability to navigate and interpret complicated real-world clinical information effectively.

However, the relatively strong performance of LLMs, particularly in organ-body part predictions, is noteworthy. High precision in this category, demonstrated by both Claude-3-Haiku and GPT-4, suggests that when these models provide positive predictions, they can be highly reliable. Accordingly, there is potential for LLMs to augment clinical decision-making in specific areas, particularly those involving anatomical knowledge [23, 25]. At the same time, the models’ lower recall rates, especially in medical test justification, raise concerns about potential missed diagnoses. This limitation could have significant implications for patient care if LLMs were implemented without adequate human oversight. The balance between precision and recall, as reflected by the F1 scores, consistently favored independent experts, reaffirming the importance of evidence-based clinical guidelines for tasks requiring nuanced medical judgment [16, 34].

While the independent experts’ assessments in this study were based on consensus, future research could benefit from comparing single expert performance to model-based referrals in real-life settings. This approach would not only provide additional valuable insights but also address a key limitation of the current study: the inconsistencies and missing data in the clinicians’ referrals dataset (see below under ‘Limitations’). Improving data quality is crucial for enhancing LLM performance and generalizability. Developing structured referral templates and collecting comprehensive contextual data could provide cleaner benchmarks for model evaluations. These improvements would allow a more nuanced understanding of how LLMs perform in relation to individual expert assessments under varying real-world conditions, potentially uncovering new avenues for AI integration in clinical workflows.

The broader implications of these findings emphasize the opportunities and challenges in integrating AI into radiology. LLMs show promise in reducing radiologist workload. However, practical adoption of these tools requires overcoming significant challenges, including ensuring the interpretability of recommendations, mitigating biases, and maintaining compliance with regulations such as the GDPR. Additionally, robust evaluation frameworks are needed to validate model performance across diverse populations and imaging modalities, ensuring that AI tools align with the standards of clinical practice [34–36]. Collaborations bringing together clinicians, ethicists, engineers, and researchers will be crucial to address the technical, practical, and societal challenges of integrating LLMs responsibly into healthcare.

Beyond these challenges, another key consideration is the trust that the scientific and clinical communities place in AI evaluation tools. The variability observed in GPTZero’s AI-generated text detection aligns with broader concerns regarding the reliability of AI-driven assessments. This raises an important question about the confidence placed in AI tools, particularly in radiology, where AI is increasingly integrated into clinical workflows. A recent study highlights the evolving role of AI in radiology, revealing patterns of AI adoption and clinical implications. These findings underscore the necessity of establishing robust validation frameworks to ensure that both clinicians and researchers can rely on AI-generated outputs with confidence. Understanding the limitations of AI assessment tools is crucial not only for text evaluation but also for clinical decision support systems, where trust directly impacts patient care and diagnostic accuracy. The radiological community must continue to critically evaluate these tools, ensuring their alignment with evidence-based medical practice and regulatory standards.

Finally, an emerging consideration in AI-driven clinical decision support is the potential role of alternative models such as DeepSeek. With its reported lower production costs and reduced environmental impact, DeepSeek presents an interesting option for radiology applications. However, beyond efficiency and sustainability, the practical implementation of such models in clinical workflows requires careful scrutiny. Key concerns include data security, regulatory compliance, and the ability to integrate with existing hospital systems. Unlike traditional AI models, which operate within strict data governance frameworks, newer models must undergo rigorous validation to ensure their outputs align with medical guidelines and patient safety requirements. Moreover, regulatory challenges such as adherence to GDPR and HIPAA standards further complicate the widespread adoption of such technologies in healthcare. Future research should explore whether models like DeepSeek can provide clinically useful and explainable recommendations while maintaining robust data security and compliance with medical regulations.

## Limitations

This study had several important limitations that should be considered. First, while the large multi-center dataset aimed to be representative, variability in local documentation practices, guidelines, and exam ordering behaviors across healthcare systems introduces uncertainty. Standardizing referral templates and developing criteria tailored to local practices while still capturing individual factors could help reduce variability. Electronic health records encouraging structured documentation could also improve consistency without oversimplifying complex cases.

Second, missing data reduced the sample available for some comparisons and potentially biased results. Imputation techniques and aggregation of related variables could help address missing values respectfully. Collecting additional referral details not captured in this CT-focused study would provide a more comprehensive picture of imaging utilization.

Third, artificial constraints were introduced in the LLM prompts to exclude MRI scan recommendations, which were outside the scope of this CT-focused study. While necessary to ensure a fair comparison with the reference standard and expert benchmarks, this modification may have limited the models’ ability to provide fully optimized suggestions across all imaging modalities. Future studies could evaluate LLMs in datasets that include a broader range of imaging options, allowing for a more comprehensive assessment of their decision-making capabilities without such constraints.

Finally, models were only assessed on CT justification rather than a clinician’s full scope of responsibilities, limiting conclusions about other tasks. Evaluating models on additional clinically relevant tasks like follow-up recommendations or test interpretation could provide more robust validation of their capabilities. Assessing rationales for recommendations through explainable LLMs could also promote reliable integration into practice.

## Conclusions

This study presents a rigorous evaluation of LLMs and a guideline-based clinical decision support system for CT referral justification. The results demonstrate the advantages of evidence-based guidelines for this specialized task but also indicate LLMs’ performance can be enhanced through targeted data refinement and model optimization. Looking ahead, a hybrid approach leveraging both approaches may best optimize diagnostic imaging appropriateness and utilization.

While this study demonstrates the potential of LLMs in specific aspects of CT referral justification, their current limitations underscore the continued importance of human expertise. LLMs should be developed as complementary tools to augment clinical decision-making rather than replace human judgment. Future efforts must prioritize optimizing LLMs for specific clinical tasks, addressing data quality issues, and creating robust evaluation frameworks to ensure their safe and effective integration into real-world practice.

## Abbreviations

ACR: American College of Radiology
AI: Artificial intelligence
Claude-3 Haiku: A specific configuration of the Claude-3 model by Anthropic which is optimized for high-speed inference
CT: Computed tomography
ESR iGuide: European Society of Radiology’s clinical decision support system for imaging referral guidelines
GDPR: General Data Protection Regulation
GPT-4: Generative pre-trained transformer 4
IRBs: Institutional review boards
LLMs: Large language models
